# Stability of influenza viruses in the milk of cows and sheep

**DOI:** 10.1099/jgv.0.002257

**Published:** 2026-05-07

**Authors:** Jenna Schafers, Caroline J. Warren, Jiayun Yang, Junsen Zhang, Sarah J. Cole, Jayne Cooper, Karolina Drewek, Natalie McGinn, Mehnaz Qureshi, Scott M. Reid, Nunticha Pankaew, Wenfang Spring Tan, Sarah K. Walsh, Ashley C. Banyard, Ian Brown, Paul Digard, Munir Iqbal, Joe James, Thomas P. Peacock, Edward Hutchinson

**Affiliations:** 1 Roslin Institute, Easter Bush Campus, The University of Edinburgh, Midlothian EH25 9RG, UK; 2 Influenza and Avian Virology Workgroup, Department of Virology, Animal and Plant Health Agency-Weybridge, Woodham Lane, New Haw, Addlestone, Surrey KT15 3NB, UK; 3 The Pirbright Institute, Ash Road, Woking, Surrey GU24 0NF, UK; 4 MRC-University of Glasgow Centre for Virus Research, 464 Bearsden Road, Glasgow G61 1QH, UK; 5 WOAH/FAO Reference Laboratory for Avian Influenza, Animal and Plant Health Agency-Weybridge, Woodham Lane, New Haw, Addlestone, Surrey KT15 3NB, UK

**Keywords:** dairy, food safety, H5N1, influenza virus, milk

## Abstract

In late 2023, H5N1 high-pathogenicity avian influenza virus (HPAIV) started circulating in dairy cattle in the USA. High viral titres were detected in milk from infected cows, raising concerns about onward human infections. Although pasteurisation was shown to effectively inactivate influenza viruses in milk, unpasteurised milk still poses a risk of infection, both from occupational exposure in dairies and from the consumption of raw milk. We therefore assessed how long influenza viruses could remain infectious in milk without heat inactivation. We examined the stability of a panel of influenza viruses in milk, including a contemporary H5N1 HPAIV and a variety of other influenza A and D viruses. We incubated viruses in cows’ milk under laboratory conditions: at room temperature to simulate exposure in dairies and at 4 °C to simulate exposure to refrigerated raw milk. Following an isolated report of H5N1 viral RNA detection in milk from a sheep in the UK, we also carried out similar experiments with a laboratory strain of influenza A virus in sheep’s milk. Although the survival of influenza viruses in milk was variable, we consistently found that, under laboratory conditions, substantial viral infectivity remained over periods when people might reasonably be exposed to infected milk – for over a day at room temperature and for more than 7 days when refrigerated. Our results highlight the zoonotic risk of H5N1 HPAIV in raw milk from infected animals and reinforce the importance of taking measures to mitigate this risk.

## Data Availability

The data needed to reproduce the findings and figures reported are available at the Open Science Framework (https://osf.io/gwezf/).

## Introduction

In March 2024, following reports of significantly reduced milk yield, high-pathogenicity avian influenza virus (HPAIV) of the H5N1 subtype was detected circulating among dairy cattle in the USA. The virus soon became widely distributed in dairy cattle across the country, and subsequent phylogenetic analysis suggested that the first introduction into cattle had occurred in late 2023 [1]. The outbreak was unexpected: although cattle can be experimentally infected with influenza A viruses (IAVs, the genus to which HPAIVs belong), they were not known to be natural hosts of these viruses [2]. Also unexpected was the mode of transmission – while influenza viruses in mammals are primarily respiratory infections, in cattle the virus was shed at extremely high levels in milk [3,6]. At the time of writing, H5N1 HPAIV has been detected in over a thousand herds of cattle across the contiguous USA, as well as in dozens of infected dairy workers and in several cryptic infections of people reporting no direct contact with infected animals [6]. An isolated outbreak on a dairy farm in the Netherlands has also recently been reported [7]. The genetic material of H5N1 HPAIV has been detected in consumer dairy products across the USA, with ∼20% of retail milk samples testing positive for viral RNA in some affected areas [8]. Although studies have confirmed that pasteurisation effectively inactivates H5N1 HPAIV in milk [9,18], unpasteurised (‘raw’) milk may pose an infection risk, through both occupational exposure and consumption.

Occupational exposure to H5N1-contaminated milk is a risk for dairy workers in affected areas, with one study reporting an H5 seroprevalence of 7% in dairy workers [19]. Multiple human infections have now been associated with dairy work in the USA [6], such as a dairy worker who developed unilateral conjunctivitis following a splash of milk into the eye, acquired during milking without the use of eye or face protection, and was subsequently diagnosed as infected with H5N1 HPAIV from cattle [20]. In addition, on multiple dairy farms at different stages of the outbreak, environmental sampling has detected H5N1 genetic material in settings where milk handling occurs [21].

The risks posed by consuming raw milk, or milk products, that are contaminated with H5N1 are harder to assess. IAVs can remain infectious in milk and in some milk products: adding influenza viruses to milk only marginally reduced infectivity in *in vitro* assays [9], and the virus retained its infectivity for over 60 days in cheese made from unpasteurised cow’s milk [22]. Consumption of H5N1-contaminated milk has been identified as a direct infection route in certain animals. Over 50% of cats on a Texas dairy farm fell ill after consuming raw colostrum and milk from cows infected with H5N1 [3], and H5N1-contaminated milk was infectious in direct oral inoculation of mice and was a plausible route of transmission between lactating mice or ferrets and their pups [17,23, 24]. However, it should be noted that the specific details of how different animals eat, and of experimental infections, mean that none of these studies are an ideal proxy for human consumption of milk or dairy products. At the current time, the infectious dose of H5N1 through the consumption of milk by any animal, including humans, remains unknown. What we do know is that milk from H5N1-infected cattle can contain virus at extremely high infectious titres [3,25], and repeated observations in the USA of human infections by bovine H5N1 in people without a clear history of exposure to infected animals suggest that consumer exposure to H5N1 in raw milk may pose a meaningful risk of infection. This is particularly noteworthy given surveys indicating that ∼1–2% of the US population consumed raw milk products on a weekly basis between 2014 and 2022 [26,28], and that, even following the detection of H5N1 in milk, a 2025 survey found that 18% of respondents considered raw milk to be as safe as or safer than pasteurised milk, while 25% reported being unaware of the risks associated with raw milk consumption [29].

Influenza virus particles do lose their infectivity over time, and, given that unpasteurised (‘raw’) milk could contain H5N1 influenza virus, it was important to determine how long contaminated milk could remain infectious. In the dairy industry, H5N1 HPAIV might be found in milk present on surfaces across dairies, including milking and milk transport equipment (upstream of pasteurisation, if this is carried out). This could pose a transmission risk to dairy farm workers, by direct contact or through aerosol exposure during milking, transport or cleaning [30]. In these settings, spilt milk is likely to remain at ambient temperatures until it is removed by cleaning. During this time, it may dry out and be exposed to a variety of environmental contaminants and to UV light. In contrast, in settings where raw milk is consumed it is more likely to have been refrigerated, to be largely free of environmental contaminants and to be stored away from UV light sources, both at the point of purchase and in domestic refrigerators.

In this paper, we assessed the risk that influenza viruses could remain stable in unpasteurised milk. For a panel of influenza viruses, including H5N1 HPAIV, we assessed how rapidly infectivity was lost in cow’s milk at both room temperature (simulating dairy farm environments) and 4 °C (simulating customer refrigeration). In response to a recent isolated case of a lactating sheep in the UK shedding H5N1 viral RNA in its milk [31], we also assessed the stability of an influenza virus in sheep’s milk. Our findings show that IAV stability in milk is highly variable but, in the absence of other inactivating factors such as UV light, detergents or desiccation, H5N1 and other IAVs can remain infectious in milk for over a day at room temperature and more than 7 days at 4 °C – essentially, for the longest period over which milk might plausibly be left in each setting before being disposed of. Our results suggest that H5N1 HPAIV in the unpasteurised milk of infected cows or other dairy animals could serve as a source of H5N1 HPAIV exposure, both when present on surfaces in dairies and when sold for human or animal consumption.

## Methods

### Cells and viruses

Experiments using the IAV strain A/Puerto Rico/8/1934 (H1N1; ‘PR8’) and its derivatives were conducted under biosafety containment level 2 (CL2) conditions. The PR8 derivative virus PR8-H5N1 was a reassortant of PR8 with the haemagglutinin (HA) and neuraminidase (NA) genes from A/dairy cattle/Texas/24-008749-001-original/2024 (H5N1) (GISAID accession EPI_ISL_19014384). To permit handling of this virus under CL2 conditions, the polybasic cleavage site of the H5 HA segment was modified to a monobasic site. The HA and NA genes were then synthesised by GenScript and inserted into the pHW2000 vector. Virus rescue, using PR8 genes for the remaining six segments, was performed using the eight-plasmid bidirectional pHW2000 reverse genetics system in 293 T cells as previously described [32]. To produce working stocks, PR8 was propagated on Madin–Darby Canine Kidney (MDCK) carcinoma cells, and PR8-H5N1 was propagated in 9- to 10-day-old embryonated chicken eggs.

Additional viruses handled at CL2 were the IAVs A/Duck/Singapore/97 (H5N3; provided by Professor Wendy Barclay, Imperial College London) and A/wild duck/Italy/17VIR6926-1/2017 (H5N2; provided by Dr Isabella Monne, Istituto Zooprofilattico Sperimentale delle Venezie) and the influenza D virus D/bovine/France/5920/2014 (IDV; provided by Dr Mariette Ducatez, Université de Toulouse), all of which were propagated in MDCK cells.

Work using A/chicken/Wales/053969/2021 (H5N1), an H5N1-2021 clade 2.3.4.4 HPAIV derived from a UK outbreak event and representative of the UK/European epizootic season in 2021, was carried out at Specified Animal Pathogens Order (SAPO) containment level 4. The virus was propagated for 2 days in 9- to 10-day-old specified-pathogen-free embryonated eggs.

### Virus titration

For CL2 experiments, viral infectivity was quantified using a plaque assay on MDCK cells. Virus samples were diluted in tissue culture medium to mitigate cytopathic effects associated with undiluted milk.

Plaques were visualised either by direct staining of the monolayer or, for IDV, by immunocytochemistry with a custom sheep polyclonal antibody against IDV nucleoprotein (NP; available from www.influenza.bio: third bleed, 1:500), followed by an Alexa Fluor™ 568 donkey anti-sheep secondary antibody (Thermo, 1:1,000) and a DAPI counterstain (1:500). Fluorescent signals were visualised using a Celigo imaging cytometer (Nexcelom).

For work at SAPO containment level 4, virus titration and assessment in raw milk were undertaken in the presence of the antimicrobial bronopol, as described previously [9].

### Stability assay

Under CL2 conditions, virus stocks were diluted 1:10 (v/v) in test solutions. These were either PBS or milk. Cow’s milk samples were either processed (homogenised and pasteurised milk, with whole milk (4% w/v fat), purchased from commercial suppliers in the UK, where no cases of bovine IAV had been confirmed at the time of the study) or raw (obtained directly from cows in a herd managed by the University of Edinburgh and used without prior processing). Pasteurised sheep’s milk was purchased from a commercial supplier in the UK. Milk was either used on the day of acquisition or kept refrigerated at 4 °C or frozen at −20 °C to prevent spoilage prior to experimentation. To test viral stability, diluted virus was aliquoted into tubes with sealed lids to prevent evaporation. These were placed in a sealed polystyrene box at room temperature or 4 °C and left for the indicated time.

For work at SAPO containment level 4, virus titration and assessment in milk were undertaken as described previously [9].

## Results

**Fig. 1.**
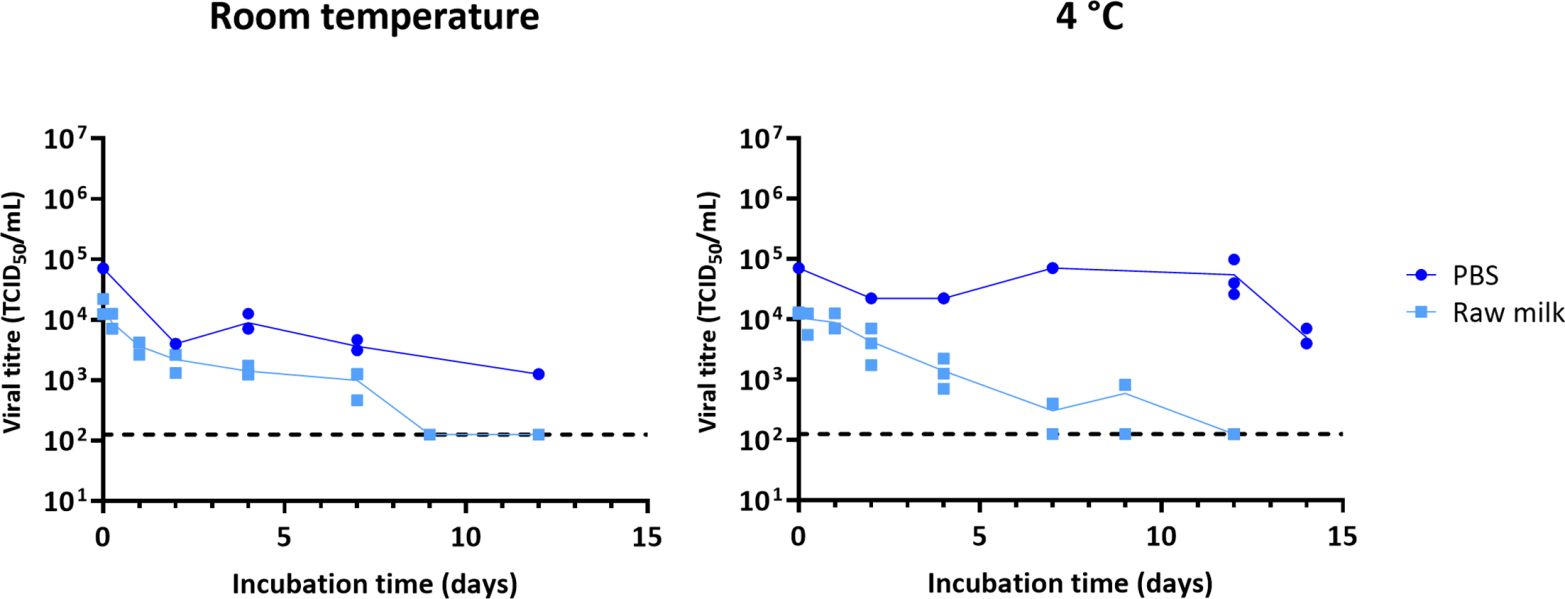
Stability of H5N1 HPAIV in milk. H5N1 HPAIV was mixed with unpasteurised cow’s milk or PBS and either incubated at room temperature (~20 °C) or chilled (4 °C) for the indicated times. Infectivity was measured by TCID_50_. Data points show three independent repeats, with lines connecting the mean values. Limit of detection = 126 TCID_50_ ml^−1^.

To assess the survival of H5N1 HPAIV in milk, we initially tested the WT strain A/chicken/Wales/053969/2021 (AIV08/AH genotype) under SAPO containment level 4 conditions. The virus was mixed at a ratio of 1:10 (v/v) with unpasteurised (‘raw’) milk. It was incubated in closed tubes to prevent evaporation, which were stored within sealed polystyrene boxes to stabilise temperature and prevent UV light inactivation. The mixture was held at either room temperature (~20 °C) or chilled (~4 °C) for up to 12 days. In parallel, the virus was mixed 1:10 with PBS and stored under identical conditions. Viral infectivity was assessed using 50% tissue culture infectious dose (TCID_50_) assays. The H5N1 virus slowly lost infectivity over time in milk, both at room temperature and under refrigerated conditions. Under either condition, infectious titres remained detectable in raw milk for over 7 days (Fig. 1).

**Fig. 2.**
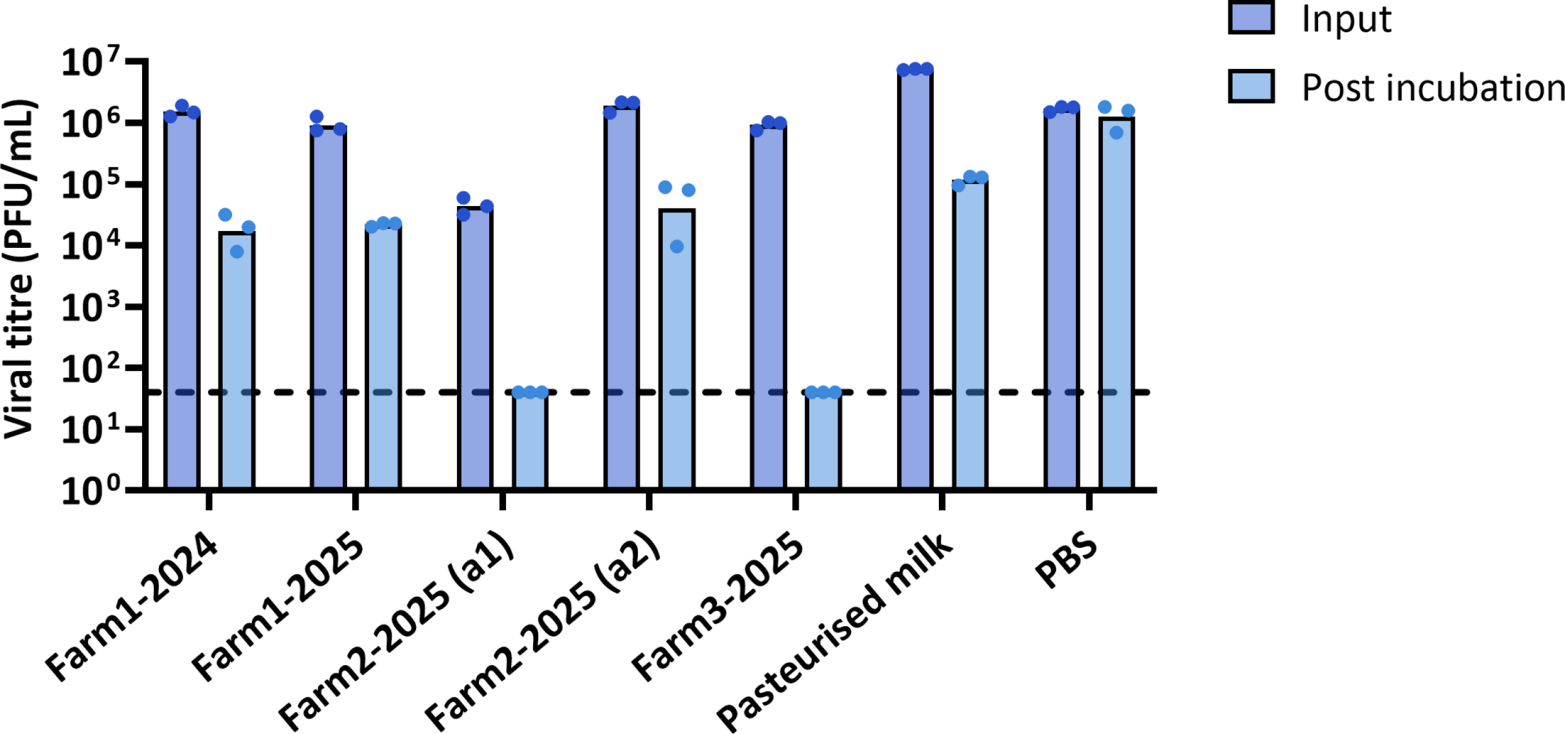
Stability of influenza virus in different sources of milk. PR8 was mixed 1:10 with unpasteurised cow’s milk collected from the same farm at different time points (a1 and a2 are different aliquots), with pasteurised whole milk, or with PBS, and either sampled immediately or incubated chilled (4 °C) for 7 days. Infectivity was measured by plaque assay. Boxes indicate the mean values, and data points represent three independent repeats. Limit of detection = 40 PFU ml^−1^.

To assess whether the source of milk contributed to the observed differences in viral stability, we next tested virus survival in raw milk collected from different farms and at different times, using the laboratory IAV strain A/Puerto Rico/8/1934 (PR8; Fig. 2). After 7 days at 4 °C, viral infectivity was titrated by plaque assay. Viral stability varied between milk sources, and even among different aliquots derived from the same source. To standardise further experiments, we tested pasteurised whole milk in the same assay and found that viral stability in pasteurised milk was comparable to that of the raw milk samples.

**Fig. 3.**
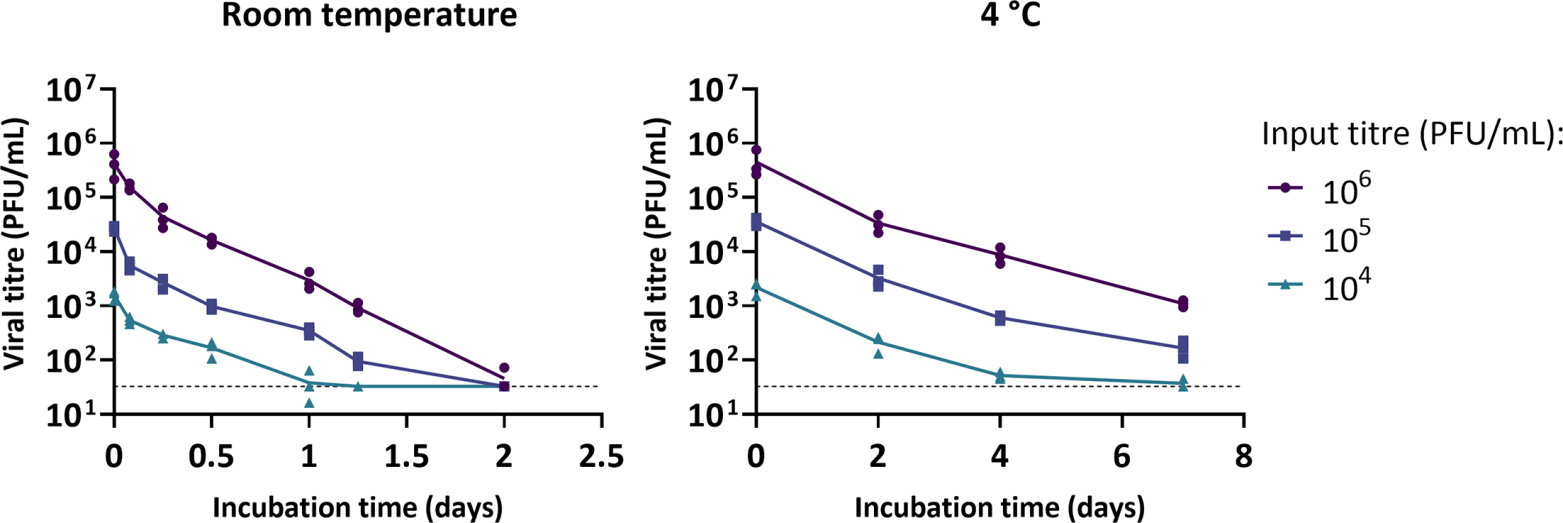
Stability of influenza virus in whole milk at different input viral titres. PR8 was diluted in PBS, then mixed 1:10 with pasteurised whole cow’s milk to give the final titres shown before incubation at room temperature (~20 °C) or chilled (4 °C) for the indicated times. Infectivity was measured by plaque assay. Data points show three independent repeats, each determined as the average of two technical replicates, with lines connecting the mean values. Limit of detection = 33 PFU ml^−1^.

We next investigated whether viral concentration could influence stability by mixing a stock of PR8 with cow’s milk (pasteurised whole milk purchased in the UK) to final titres ranging from 10^4^ to 10^6^ plaque forming units (PFU) ml^−1^ and incubating at either 4 °C or room temperature (Fig. 3). Viral infectivity declined at comparable rates regardless of the titre, indicating that, across this range of concentrations, decay kinetics were largely independent of viral concentration. Even at a relatively low input titre (10⁵ PFU ml^−1^), infectious virus remained detectable for more than 1 day at room temperature (~20 °C) and for at least 7 days when chilled (4 °C).

**Fig. 4.**
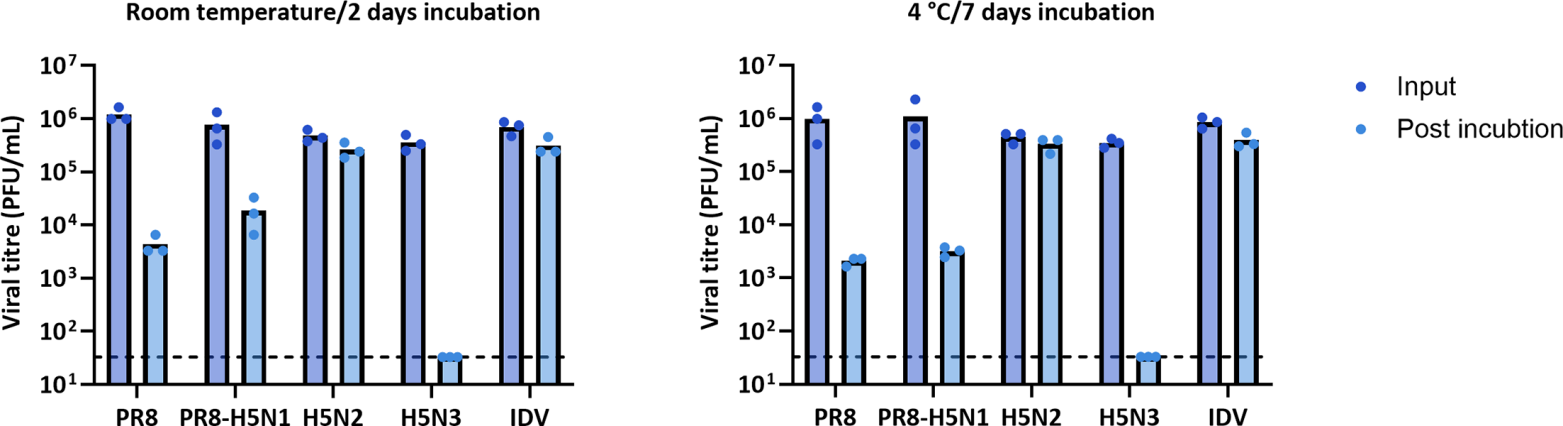
Stability of a panel of influenza viruses in milk. Viruses were titrated in media or mixed with pasteurised whole cow’s milk and incubated at room temperature (~20 °C) or chilled (4 °C) for the indicated times. Infectivity was measured by plaque assay. Data indicate three independent repeats, each calculated as the average of duplicate technical replicates (a single replicate for IDV), with boxes indicating the mean values. Limit of detection = 33 PFU ml^−1^.

To assess whether this stability in milk was consistent across other influenza viruses, we tested a panel of additional strains (Table 1). This included a reassortant influenza virus with the external genes of a bovine H5N1 clade 2.3.3.4b (with the HA ‘de-engineered’ to replace the multibasic cleavage site, which is associated with high pathogenicity, with a single-basic cleavage site) and the internal genes of PR8, allowing its use at biosafety containment level 2. In addition, the panel included PR8, two low-pathogenicity avian IAVs and an influenza D virus that naturally infects cattle. These samples were incubated in cow’s milk (pasteurised whole milk purchased in the UK) or PBS, and infectivity was measured by plaque assay (Fig. 4). Although we observed substantial variation in viral stability (Fig. S2), a consistent observation was that, for most strains, infectious virus was detectable in milk after at least 2 days at room temperature and more than 7 days at 4 °C.

**Table 1.** Influenza viruses used in the study

Short name	Strain name and details
H5N1	A/chicken/Wales/053969/2021 (H5N1), an HPAIV
PR8	A/Puerto Rico/8/1934 (H1N1), a laboratory-adapted IAV
PR8-H5N1	A reassortant virus with the internal gene of A/Puerto Rico/8/1934 (H1N1), and the HA and NA genes of A/dairy cow/Texas/24–008749001-original/2024 (H5N1)
H5N2	A/wild-duck/Italy/17VIR69261/2017 (H5N2), a low pathogenicity avian influenza virus
H5N3	A/Duck/Singapore/97 (H5N3), a low pathogenicity avian influenza virus
IDV	D/bovine/France/5920/2014, a separate genus of influenza virus that naturally infects cattle

**Fig. 5.**
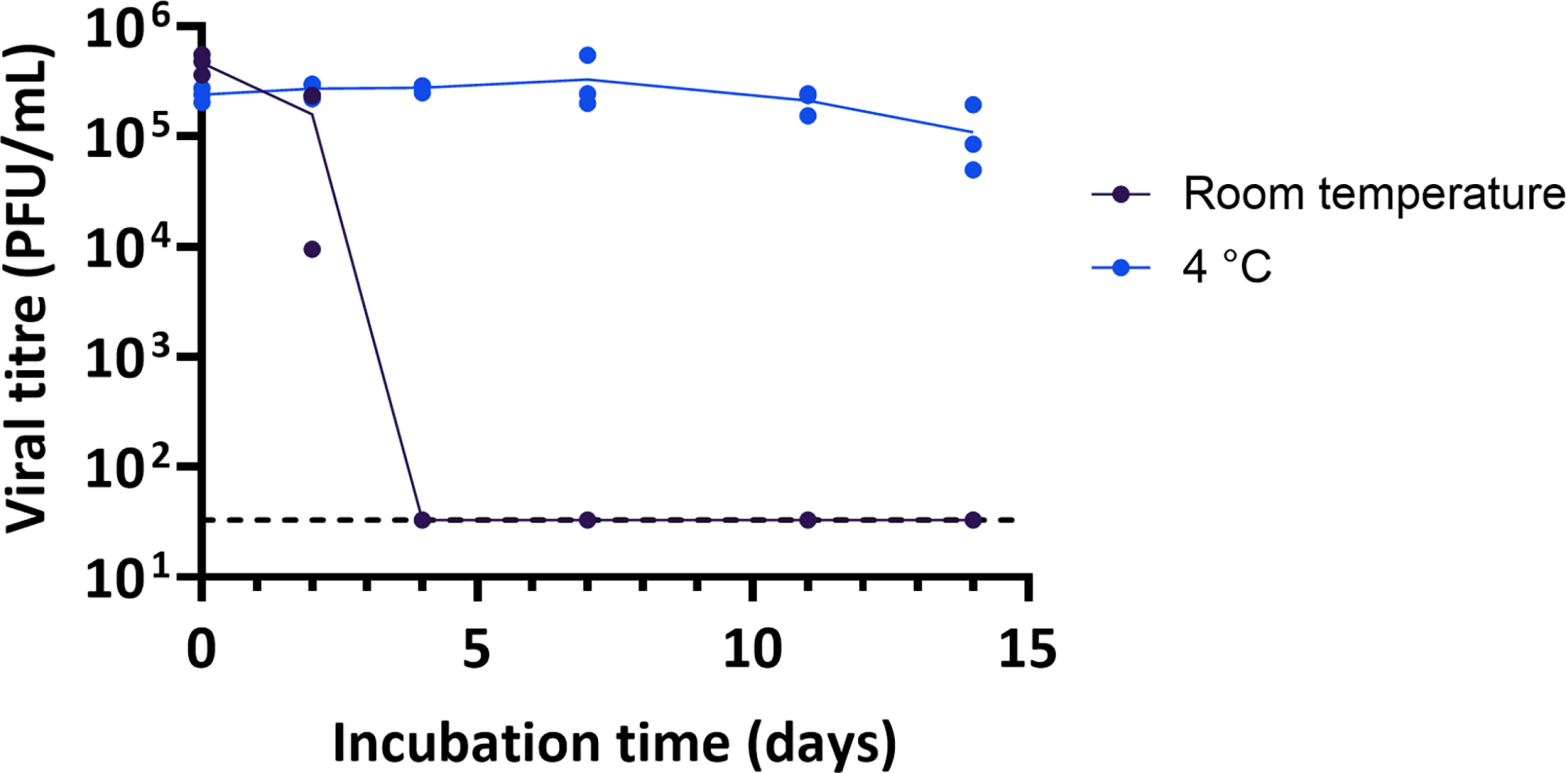
Stability of influenza virus in sheep’s milk. PR8 was mixed with pasteurised whole sheep’s milk and either incubated at room temperature (~20 °C) or chilled (4 °C) for the indicated times. Infectivity was measured by plaque assay. Data points show three independent repeats, each calculated as the average of two technical replicates, with lines connecting the mean values. Limit of detection = 33 PFU ml^−1^.

Finally, in response to an isolated case in the UK in which milk from a lactating sheep was found to be positive for H5N1 HPAIV viral RNA [31], we tested viral stability in sheep’s milk. In sheep’s milk, PR8 remained infectious for more than 7 days at 4 °C and at least 2 days at room temperature (Fig. 5), suggesting that its stability in sheep’s milk was broadly similar to that in cow’s milk.

## Discussion

In response to the detection of H5N1 HPAIV in milk from infected dairy cattle in the USA, we investigated the stability of influenza viruses in milk. We considered two scenarios – milk spilt in a dairy and left at ambient temperatures prior to cleaning, and raw milk refrigerated for consumer use. We tested the infectivity of influenza virus particles in milk under laboratory conditions, recognising that, particularly in the case of a dairy, a large number of additional environmental factors could inactivate viruses to a variable and unpredictable degree. These include (though are not limited to) UV irradiation, dehydration, changes in pH due to bacterial growth in the milk, adsorption to surfaces and the introduction of substances including detergents and the residues of cleaning products. Our experimental design deliberately excluded these non-thermal factors: we mixed viruses with milk in small, consistent volumes in sealed containers stored in the dark. Other than for the data shown in Figs 1 and 2, we attempted to reduce the effect of microbial contamination by using pre-pasteurised milk. Our experiments therefore aimed to model the ‘worst-case scenario’ for the persistence of viral infectivity in milk and should be seen as providing an upper-bound estimate for viral survival under real-world conditions.

Under our experimental conditions, H5N1 HPAIV was less stable in milk than in buffered media, but it still remained detectably infectious in milk for over 7 days at both room temperature and 4 °C (Fig. 1). We tested whether viral stability could be influenced by the source of milk and confirmed that this was the case – indeed, we even observed differences between aliquots of milk derived from the same source (Fig. S1, available in the online Supplementary Material). This is consistent with other studies of the stability of influenza viruses in milk [16,33,36] (Table 2). Milk is a non-standardised biological medium, whose composition is influenced by differences both within and between herds, as well as by environmental conditions once harvested [37]. Even after pasteurisation, milk remains non-sterile and undergoes pH changes over time due to bacterial fermentation and lactic acid production, which would likely impact virus–milk interactions [37]. Some studies have also suggested that components of milk can directly alter the infectivity of influenza viruses [38,39]. Because of these sources of variation, we felt it inappropriate to estimate a rate of decay for influenza virus infectivity in milk, and instead based our risk assessment on a binary assessment of whether infectivity could be detected after incubation for a fixed period at a fixed temperature.

**Table 2.** Comparison of studies of influenza virus stability in milk

Study	Virus	Milk type	Incubation condition	Survival time milk	Detection method	Note
This study	2.3.4.4b+IAVs+IDV	Pasteurised or unpasteurised cow’s milk and pasteurised sheep’s milk	4 °C; RT	4 °C: variable (from 1 to more than 14 days); RT: at least 1 day	Plaque assay; TCID_50_	Bulk milk
Le Sage *et al*. [33]	A/dairy cattle/TX/8749001/2024 (H5N1) A/California/07/2009 (H1N1)	Unpasteurised milk	21 °C, 23.6–25 °C 70% relative humidity	Inflation liners: at least 3 h; stainless steel: at least 1 h	TCID_50_	On surface (inflation liners and stainless steel)
Kaiser *et al*. [34]	A/bovine/OH/B24OSU-342/2024 (2.3.4.4b)	Unpasteurised milk	Bulk milk: 4 °C; 22 °C On surface: 4 °C with 80% relative humidity; 22 °C with 65% relative humidity	Bulk milk: 4 °C, more than 7 days; 22 °C, more than 7 days On surface: 4 °C, more than 7 days; 22 °C, at least 2 days	TCID_50_	On surface (stainless steel or polypropylene) and bulk milk
Caceres *et al*. [16]	A/Puerto Rico/8/1934 (H1N1) A/Vietnam/1203/2004 (H5N1) A/turkey/Indiana/3707-003/2022 (H5N1) A/Texas/37/2024 (H5N1)	Unpasteurised and pasteurised milk	4 °C RT 37 °C	4 °C: more than 4 days; RT: more than 4 days; 37 °C: variable (from 1 to more than 4 days)	TCID_50_	Bulk milk
Zulli *et al*. [35]	A/Puerto Rico/8/1934 (H1N1)	Unpasteurised milk	4 °C	At least 5 days	TCID_50_	Bulk milk
Hu *et al*. [36]	A/WSN/1933 (A/H1N1), A/chicken/CHN/Cangzhou03/2023 (A/H5N1), D/bovine/CHN/JY3002/2022 (D/Yama2019)	Pasteurised milk	4 °C	At least 6 days	TCID_50_	Bulk milk
Guan *et al*. [44]	Multiple H5N1 strains isolated from dairy cows A/blue-winged teal/Wisconsin/402/1983 (H4N6) A/swine/Ohio/09SW73E/2009 (H3N2) A/equine/London/1416/1973 (H7N7) A/Isumi/UTKK001-1/2018 (H1N1) PR8-H5N8 (A/Puerto Rico/8/1934+A/Astrakhan/3212/2020)	Unpasteurised milk	4 °C	Infected cow’s milk: more than 22 weeks; spiked in milk: at least 2 weeks	Plaque assay	Bulk milk and infected cow’s milk
Lenz-Ajuh *et al*. [45]	A/cattle/Texas/063224-24-1/2024 (H5N1) A/duck/Hokkaido/Vac-1/2004 (H5N1)	Unpasteurised milk	4, 21, 37 °C	4 °C: more than 4 weeks; 21 °C: more than 4 weeks; 37 °C: at least 2 weeks	TCID_50_, FFU	Bulk milk
Nooruzzaman *et al*. [46]	A/cattle/Texas/063224-24-1/2024 (H5N1)	Unpasteurised milk	4, 20, 30, 37 °C	Infected cow’s milk and spiked milk: 4 °C, 42 days; 20 °C, 7 days; 30 °C, 5 days; 37 °C, 1 day	TCID_50_	Bulk milk and infected cow’s milk

FFU, focus forming unit; PFU, plaque forming unit; RT, room temperature; TCID_50_, 50% tissue culture infectious dose.

To minimise the variation introduced by microbial growth in milk, we carried out our remaining experiments by spiking virus into pre-pasteurised milk, in which we found the virus decayed at rates comparable to those in raw milk (Fig. 2). We found that the decay of viral infectivity in milk was not obviously affected by the concentration of virus, and that detectable virus could be found for over a day at room temperature and a week at 4 °C even for the lowest input titres tested (Fig. 3). By considering a panel of influenza viruses, we found that the influenza virus infectivity in milk typically persisted for at least a day at room temperature (~20 °C) and for 7 days or more when refrigerated (4 °C; Figs 4 and S2). Additionally, despite the mutation of the multibasic cleavage site to a monobasic site in the PR8-H5N1 virus (Fig. 4), H5N1 in Fig. 1 and the PR8-H5N1 strain exhibited broadly comparable stability in milk under similar conditions, further supporting the conclusion that the milk source, rather than viral genotype, is the primary determinant of viral stability in milk. Our findings consistently demonstrate that influenza viruses can remain infectious in milk for the time periods in which humans are likely to be exposed to milk in either an occupational or a consumer setting: for over a day at room temperature, and for over a week at 4 °C.

These findings underscore the infection risks associated with milk in areas where dairy animals are infected with H5N1 influenza viruses. At the time of writing, H5N1 is widespread in dairy cattle across the continental USA but is not yet known to be circulating in cattle in other countries [40], and the detection of H5N1 in a sheep in the UK remains an isolated incident. Importantly, pasteurisation effectively inactivates influenza viruses [10,14]. However, our data show that without pasteurisation, influenza viruses can remain infectious for an extended period in milk. Similarly, other studies have shown that in unpasteurised dairy products, such as cheese and yoghurt, influenza viruses can remain infectious for remarkable periods of time [22,41,43].

Our data suggest that when H5N1 infection is suspected, there are potential occupational and consumer infection risks due to spilled milk in dairy environments, as well as during the storage, transport and consumption of refrigerated, unpasteurised milk. In real-world scenarios, environmental factors may reduce virus stability to an unpredictable extent. However, we note that neither our study nor the other studies performed to date clearly establish an upper limit for how long influenza viruses can remain infectious in milk (Table 2). In terms of spilled or stored milk, given the extremely high titres of virus that have been reported in milk from infected cattle [3,25], even small volumes of milk could pose a significant infection risk for as long as they are likely to be encountered in the environment. Our data therefore suggest that the potential for infection from contaminated milk should be considered when planning control measures for H5N1 HPAIV in dairy settings, during milk processing and where raw milk is sold to consumers.

## Supplementary material

10.1099/jgv.0.002257 Uncited Supplementary Material 1.

## Abbreviations

BSL-2: biosafety level 2
CL2: containment level 2
HPAIV: high-pathogenicity avian influenza virus
IAVs: influenza A viruses
MDCK: Madin–Darby Canine Kidney
TCID_50_: 50% tissue culture infectious dose
