# Supplementary material for: Stability of influenza viruses in the milk of cows and sheep
Source: J Gen Virol. 2026 May 7;107(5):002257. doi: 10.1099/jgv.0.002257 (PMC13151985; doi:10.1099/jgv.0.002257)
Supplement: Uncited Supplementary Material 1. [file jgv-107-02257-s001.pdf]

Supplementary data

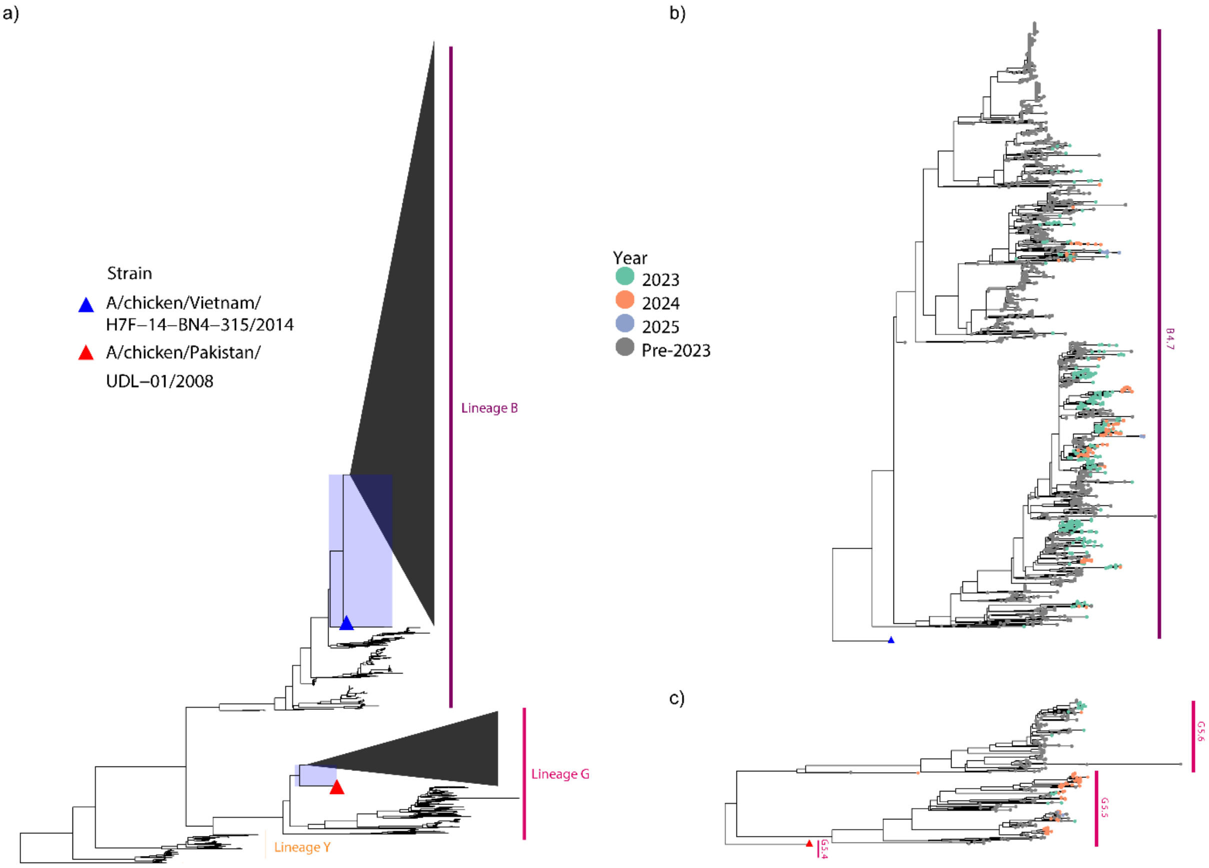

Supplementary Figure S1: Phylogenetic analysis of H9 Haemagglutinin (HA) gene

All complete HA genes of H9N2 viruses from January 2020 (around 3357 sequences) were downloaded from the GISAID EpiFlu database accessed on 14th July 2025. The dataset was aligned using Mafft v7.525 (Katoh and Standley 2013) and manually trimmed to the open reading frame using Aliview v1.26 (Larsson 2014). The trimmed alignments were used to infer maximum likelihood phylogenies using IQ-Tree v2.4.0 with the GTR substitution model (Minh, Schmidt et al. 2020). Panel a) shows the overall phylogenetic tree, highlighting the major H9N2 lineages: BJ94 like (lineage B), G1 like (lineage G) and Y439 like (lineage Y). Panels (b) and (c) show expanded views of the B4 and G5 clades which include the HA genes of Vietnam/315 H9N2 and Pakistan/UDL-01 H9N2, respectively.

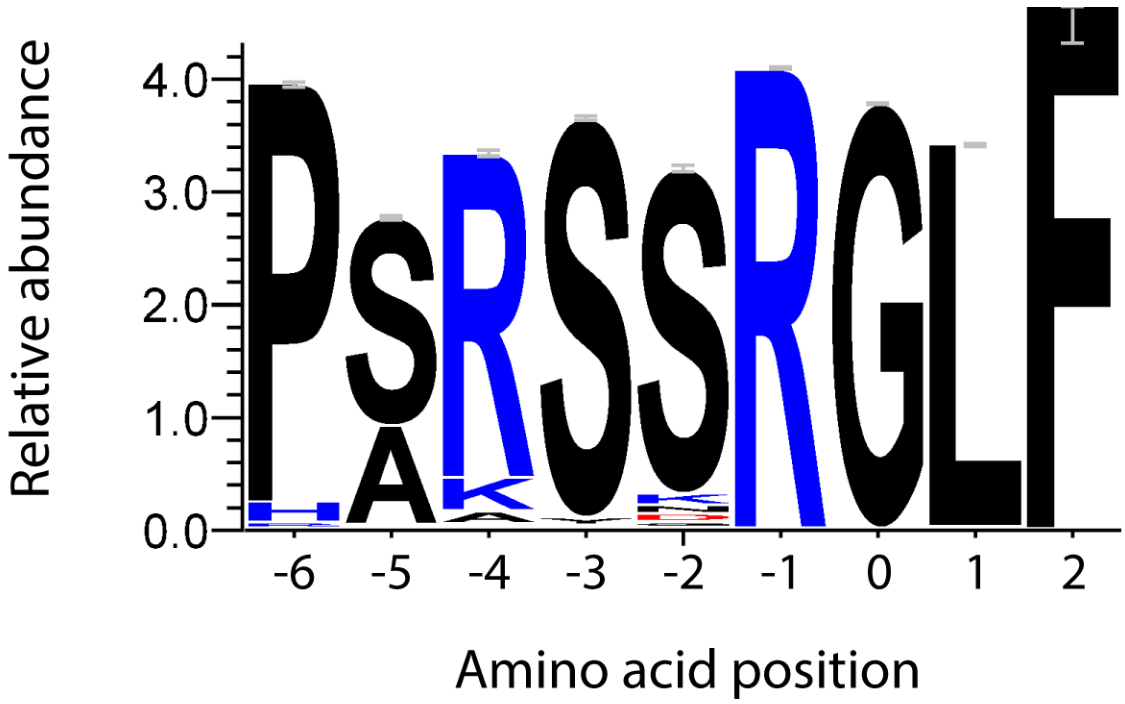

Supplementary Figure S2: Amino acid analysis of H9 Haemagglutinin (HA) cleavage site shown as a sequencing logo.

Each logo consists of a symbol or a stack of symbols representing an amino acid at the H9HA cleavage site. The overall height of the stack (shown as bits) indicates the sequence conservation at that position. The height of the symbols within each stack represents the relative frequency of each amino acid at that position. Amino acids are coloured according to their chemical properties. The analysis included 12910 H9HA sequences retrieved from GISAID. The weblogo was created using Weblogo 3 (webversion) (Crooks, Hon et al. 2004).

References:

Crooks, G. E., G. Hon, J. M. Chandonia and S. E. Brenner (2004). "WebLogo: a sequence logo generator." Genome Res **14**(6): 1188-1190.

Katoh, K. and D. M. Standley (2013). "MAFFT multiple sequence alignment software version 7: improvements in performance and usability." Mol Biol Evol **30**(4): 772-780.

Larsson, A. (2014). "AliView: a fast and lightweight alignment viewer and editor for large datasets." Bioinformatics **30**(22): 3276-3278.

Minh, B. Q., H. A. Schmidt, O. Chernomor, D. Schrempf, M. D. Woodhams, A. von Haeseler and R. Lanfear (2020). "IQ-TREE 2: New Models and Efficient Methods for Phylogenetic Inference in the Genomic Era." Mol Biol Evol **37**(5): 1530-1534.
